# Tissue‐seeking dyes for in vivo applications

**DOI:** 10.1002/smo.20240029

**Published:** 2024-10-24

**Authors:** Yuan Ma, Cuirong Qin, Yanlin Lei, Xiaodong Yang, Zuhai Lei

**Affiliations:** ^1^ School of Pharmacy Yunnan University Key Laboratory of Medicinal Chemistry for Natural Resource Ministry of Education Kunming China

**Keywords:** bone‐seeking dyes, nerve‐seeking dyes, tissue‐seeking dyes, tumor‐seeking dyes

## Abstract

In vivo optical imaging has become an invaluable tool for visualizing and monitoring biological processes in living organisms. A key component of these imaging techniques is the use of fluorescent dyes that can selectively target and label specific tissues or cell types. This review provides an overview of the current state of tissue‐seeking dyes for in vivo applications. We discuss the design principles and chemical structures of dyes that have been developed to target various tissues of interest, including tumors, nerves, bones, vasculatures, and other tissues. The review covers the photophysical properties, targeting mechanisms, and in vivo performance of these dyes. Particular emphasis is placed on dyes that have demonstrated clinical translation or have high potential for future clinical use. The review also examines the challenges and considerations in developing effective tissue‐seeking dyes, such as achieving high specificity, overcoming biological barriers and minimizing toxicity. Finally, we highlight emerging trends and future directions in the field, including the integration of tissue‐seeking dyes with advanced imaging modalities and theranostic applications. Overall, this review provides a focused summary of the current landscape of tissue‐seeking dyes and their pivotal role in advancing in vivo optical imaging and its biomedical applications.

## Graphical abstract

Fluorescent dyes that can selectively target and label specific tissues or cell types are very important in visualizing and monitoring biological processes in living organisms. This review provides an overview of the current state of tissue‐seeking dyes for in vivo applications. We discuss the design principles and chemical structures of dyes that have been developed to target various tissues of interest, including tumors, nerves, bones, vasculatures, and other tissues.

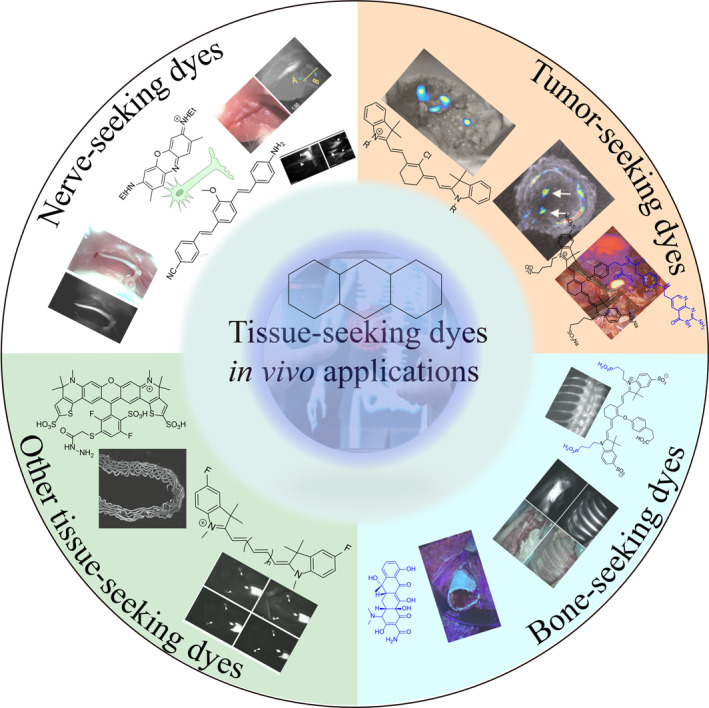

## 1 INTRODUCTION

Dyes are an ancient and vibrant class of optical chemicals.^[1]^ Nowadays, dye‐based applications are becoming more and more prevalent since researches have uncovered that many dyes possess additional properties beyond just imparting color to a substrate, for example, fluorescence imaging has emerged as a powerful technique for visualizing biological processes and diagnosing diseases at the molecular level, dye‐based photodynamic drugs have been launched on the market, and other dye‐based therapeutic modalities such as photothermal therapy or sonodynamic therapy have also received increasing attention. Among the various optical agents, such as organic small molecule dyes, quantum dots, carbon dots, rare‐earth nanoparticles, single‐walled carbon nanotube, organic small molecule dyes exhibit diverse optical properties, such as strong absorption and emission, high molar extinction coefficients, and good photostability. They can be further functionalized or modified to tune their properties, such as solubility, reactivity, and target specificity, making them versatile in various applications, including textile dyeing, biological imaging, sensing, biomedical therapy and optoelectronics. In the field of biomedical research, the in vivo imaging‐based applications and therapy applications greatly benefit from the high tissue selectivity of dyes. Therefore, tissue‐seeking dyes or the called tissue‐targeted dyes have garnered significant attention due to their ability to selectively accumulate in specific organs, tissues, or cell types. These dyes leverage unique biochemical signatures or physiological characteristics of the target tissue to achieve preferential localization and signal amplification, enabling sensitive and specific detection or accurate therapy.

The development of tissue‐seeking dyes has been driven by the need for early and accurate disease diagnosis and treatment, as well as the demand for more precise therapeutic monitoring and guidance.^[2]^ For imaging‐based applications, by selectively illuminating areas of interest, tissue‐seeking dyes offer improved contrast and signal‐to‐noise ratios compared to traditional non‐targeted dyes, facilitating the visualization of disease biomarkers, physiological processes, and pathological changes. For disease therapy, tissue‐seeking dyes offer improved therapeutic effects and reduced side effects, alongside convenient drug delivery. Various tissues are characterized by a very high degree of variability at different levels, including their structure, function, and composition. Compositionally, tissues exhibit variations in terms of the types and proportions of cells present, as well as the extracellular matrix components. These differences lay the foundation of tissue‐seeking dyes. One prominent application of tissue‐seeking dyes is in oncology, where tumor‐seeking dyes have demonstrated remarkable potential for cancer detection, staging, and surgical guidance. These dyes exploit the unique metabolic profiles, enzyme activities, or microenvironmental cues present in tumor cells or the tumor microenvironment, enabling the specific labeling of malignant tissues. Furthermore, the integration of these dyes with multimodal imaging techniques, such as magnetic resonance imaging (MRI) or positron emission tomography (PET),^[3]^ provides complementary information and enhances diagnostic accuracy. Beyond oncology, tissue‐targeted dyes have found applications in various other fields, including neuroscience, cardiovascular research, bone biology, etc. For example, nerve‐seeking dyes have facilitated the study of neuronal pathways and the detection of neurodegenerative diseases as well as nerve preservation during surgery, while bone‐targeting probes have enabled the visualization of bone metabolism and the early detection of skeletal disorders.

This review aims to provide a focused overview of the recent advances in the development and application of tissue‐seeking dyes for in vivo applications, primarily from the molecular structure perspective. We will explore the design strategies, underlying mechanisms, and key performance parameters of these dyes, highlighting their strengths, limitations, and future prospects. Additionally, we will discuss the challenges associated with translating these dyes from bench to bedside, including aspects related to biocompatibility, targeting specificity, and regulatory considerations. By elucidating the current state of the art and future directions in the field of tissue‐seeking dyes, this review seeks to foster interdisciplinary collaborations and accelerate the clinical adoption of these powerful molecular tools, ultimately contributing to improved disease management and patient outcomes.

## 2 TUMOR‐SEEKING DYES

Tumor‐seeking agents refer to compounds or molecules that have the ability to specifically target and accumulate within tumors. The development of tumor‐seeking dyes is an active area of research in oncology, as they can be utilized for imaging, diagnosis and therapeutic targeting. Usually, the key to developing effective tumor‐seeking agents is to identify unique characteristics or markers that are predominantly expressed or upregulated in tumor cells compared to normal cells. These can include specific cell surface receptors, altered metabolic pathways, or aberrant signaling cascades. By exploiting these tumor‐specific features, researchers aim to design dyes that preferentially accumulate and/or interact with cancerous tissues, leading to improved diagnostic accuracy, targeted therapeutic delivery, and enhanced treatment efficacy. However, tumor is a highly heterogeneous tissue, even ones derived from the same organ are not homogeneous. Imaging or monotherapy with one actively targeted agent is unlikely to be uniformly effective. Therefore, agents that putatively target any solid tumor tissue deserve special attention. Up to now, tumor‐seeking dyes can be roughly divided into two categories: structure inherent targeting dyes and ligand‐mediated targeting dyes.

### 2.1 Structure inherent tumor‐seeking dyes

Until now, many of the dye structures that have been reported have inherent tumor‐seeking properties. Structurally, these tumor‐seeking dyes include cyanine dyes, xanthene dyes, phthalocyanine dyes, azo dyes, Boron‐Dipyrromethene (BODIPY), etc. Due to the diversity of structures and properties, these tumor‐seeking dyes find wide applications in vivo.

#### 2.1.1 Cyanine without meso‐Cl

**FIGURE 1:**
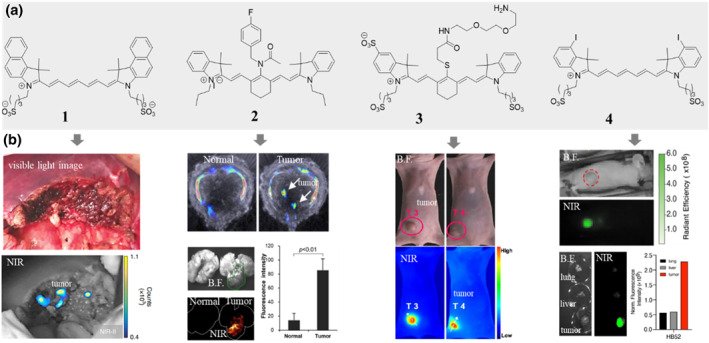
Chemical structures (a) of some selected tumor‐seeking cyanine dyes without meso‐Cl and their corresponding in vivo performances (b). ICG (1) was used for human HCC tumor imaging and the signal can be detected in the remaining tissue sections, adapted from, ref^[4]^ Copyright © 2019, The Author. In vivo multispectral optoacoustic tomography imaging and ex vivo fluorescence imaging show that compound 2 selectively accumulates in lung tumor tissue, adapted from, ref^[6]^ Copyright © 2019 American Chemical Society. compound 3 selectively accumulates in MCF‐7 tumor tissue, adapted from, ref^[7]^ Copyright © 2024 American Chemical Society. In vivo and ex vivo fluorescence imaging shows that compound 4 selectively accumulates in Hep3B tumor tissue, adapted from, ref^[8]^ Copyright © 2023 American Chemical Society.

Cyanine dyes consist of two nitrogen‐containing heterocyclic rings (typically indole or quinoline) connected by a polymethine chain. Among various cyanine dyes, Indocyanine green (ICG, **1**, Figure 1), approved by FDA in 1959, is probably the most well‐known or the dye molecule that has received the greatest amount of attention. ICG was discovered by German chemist Otto Heinrich Warburg in 1936. The compound was initially developed for use in industry and as a stain for biological tissues. In the 1950s and 1960s, researchers began exploring the medical applications of ICG. It was found to be well‐tolerated by the body and had a rapid clearance rate from the bloodstream that made it useful in various clinical settings. For example, ICG has been used in hepatic function testing to assess liver function and measure liver blood flow. It's known to accumulate in hepatocellular carcinoma (HCC) tissues after an intravenous injection, which means that ICG is an HCC tumor‐seeking dye.^[4]^ Later research suggests that ICG accumulates in HCC tissues by organic anion transporting polypeptide 1B3 (OATP1B3) mediated uptake and its subsequent multidrug resistance p‐glycoprotein (MDR3) mediated excretion into dead‐ended pseudolands or bile canaliculi.^[5]^

ICG continues to be a topic of active research, with ongoing investigations into its potential applications in cancer detection, lymphatic mapping, and drug delivery systems. Researchers are also exploring the development of new tumor‐seeking dyes based on ICG. For example, compound **2** (Figure 1), a meso‐N substituted Cy7 (vide infra) and an analogue of ICG, is identified as a lung cancer seeking dye. compound **2** was discovered by screening from a NIR dye molecule library. Both in vitro and in vivo experiments indicated that compound **2** selectively seeks tumor initiating cells (TIC) through heme oxygenase 2 (HMOX2) with a dissociation constant (K_D_) of 0.38 μM.^[6]^ Compound **3** (Figure 1), a meso‐S substituted asymmetry Cy7, has demonstrated better stability than ICG and also has some tumor‐seeking property.^[7]^ Since ICG has many modifiable sites, it is reasonable to presume that more tumor‐seeking dyes derived from ICG will emerge. Meanwhile, small changes to the structure might better retain ICG's inherent tumor‐targeting capability. For example, compound **4** (Figure 1), an iodinated ICG analog, specifically localizes to tumors in mice bearing liver cancer xenografts likely meditated by OATP1B3.^[8]^ This is an interesting result because iodine‐131 is routinely used alone or conjugated to tumor‐selective molecules as a radiopharmaceutical therapy agent, and iodine‐124 can be used for positron emission tomography (PET) imaging, as well as iodine atoms have a good heavy atom effect and are widely used in photosensitizers. Therefore, iodinated tumor‐seeking dyes have great potential in imaging and therapy applications.

We think that the research on ICG and its derivatives is paving the way for novel advancements in the field of cancer detection, imaging, and therapy. The development of tumor‐seeking dyes based on ICG holds great potential for improving diagnostic accuracy, targeted drug delivery, and personalized treatment strategies in the fight against cancer. Further studies are warranted to explore the full scope of these applications and optimize the design and effectiveness of these dyes.

#### 2.1.2 Cyanine with meso‐Cl

**FIGURE 2:**
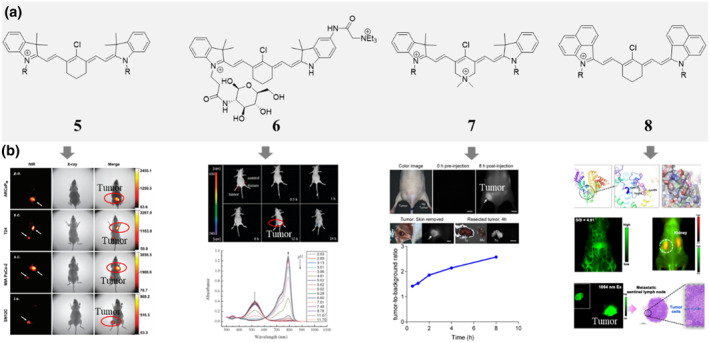
Chemical structures (a) of some selected tumor‐seeking cyanine dyes with meso‐Cl and their corresponding in vivo performances (b). NIR fluorescence images and X‐ray images show that IR783 can accumulate in different tumors, adapted from, ref^[9a]^ Copyright ©2010 AACR. In vivo NIR fluorescence imaging of compound 6 in tumor‐bearing mice and its spectra changes with pH, adapted from, ref^[15]^ Copyright © 2015 The Royal Society of Chemistry. In vivo and ex vivo fluorescence imaging tumor‐bearing mice with compound 7 and the corresponding tumor‐to‐background ratios, adapted from, ref^[17]^ Copyright © 2019 © Ivyspring International Publisher. The covalent docking between compound 8 and HSA, and the corresponding in vivo images, adapted from, ref^[19a]^ Copyright © 2024 The authors.

The typical Cy7 structure is shown in Figure 2 (compound **5**), which generally consists of a meso‐Cl substituted cyclohexenyl in the polymethine linker and two alkylated indoles at both ends. In 2010, Chung, Shi et al. discovered that some meso‐Cl substituted Cy7 dyes can selectively accumulate in tumor tissues.^[9]^ The dyes persist in tumors up to 20 days^[10]^ Interestingly, the tumor‐seeking ability of these dyes seems to have little connection with the alkyl R group (Figure 2). No matter the R is a water soluble alkyl sulfonate, an alkyl carboxyl or a lipophilic alkyl group, they can accumulate in different tumors.^[11]^ The mechanism behind this has been attributed to that tumor‐seeking dyes are uptaken by organic anion transporting polypeptides (OATPs) overexpressed on cancer cells. Since these dyes can target many solid tumors, more and more researches emerged latter.^[12]^ If the tumor‐seeking result was mainly rely on OATPs, why these dyes can persist in tumor more than 20 days? Later, research confirmed that the involvement of OATPs cannot be completely ruled out, but the weight of the evidence favors other mechanisms.^[13]^ More evidence shows that covalent albumin binding is more important to tumor uptake and persistence.[^11^, ^14^]

On the basis of the fact that Cy7 with meso‐Cl substitution has tumor‐seeking property, tumor targeted multifunctional fluorescent probe based on this framework can be expected. For example, a pH sensitive probe is constructed by connecting the meso‐Cl substituted cyclohexenyl with an alkylated indolium and a non‐N‐alkylated indolium. The resulting asymmetry Cy7 (compound **6**, Figure 2) keeps the tumor‐seeking property and is successfully used to monitor the fluctuation of pH.^[15]^ With the same strategy, other targets responsive probes or diagnostics agents or therapy agents deserve to be explored.^[16]^ Replacing the cyclohexyl methylene of Cy7 with a dialkyl ammonium fragment to get an intriguing analogue (compound **7**, Figure 2). Compared with cyclohexyl methylene Cy7, compound **7** has one more positive charge and still keeps the tumor‐seeking ability. Besides, it has better quantum yields, enhanced photostability, less cytotoxicity and complementary solubility, and is less prone to form aggregate.^[17]^ After iodinated, both Cy7 and its dialkyl ammonium analogues can be turned into a photosensitizer used for photodynamic therapy. Amusingly, the iodinated dialkyl ammonium analogue is identified to be a better preclinical candidate than iodinated Cy7 with respect to dark cytotoxicity, light/dark cytotoxicity ratio, selectivity of localization in tumors over other organs, and clearance from the plasma.^[18]^ Since the tumor‐seeking property is mainly attributed to covalent albumin binding, changing the indolium to benz[cd]indolium results in a long wavelength NIR‐II dye (compound **8**, Figure 2), which can be easily bound to albumin and then forms a biomimetic NIR‐II fluorescent protein.^[19]^

According to the reported tumor‐seeking mechanism of cyanine dyes with a meso‐Cl substitution, both Cy7 and Cy3 (generally consists of a trimethine linker and two alkylated indoles at both ends) are reasonable tumor‐seeking dyes.^[11]^ For the in vivo based applications, Cy7 maybe the better choice. The wavelength of these targeted dyes can be easily extended to the biologically preferable NIR‐II window. Further structure modifications may bring more fantastic molecules based on Cy7 for in vivo targeting imaging or therapy.

#### 2.1.3 Other structure inherent targeting dyes

**FIGURE 3:**
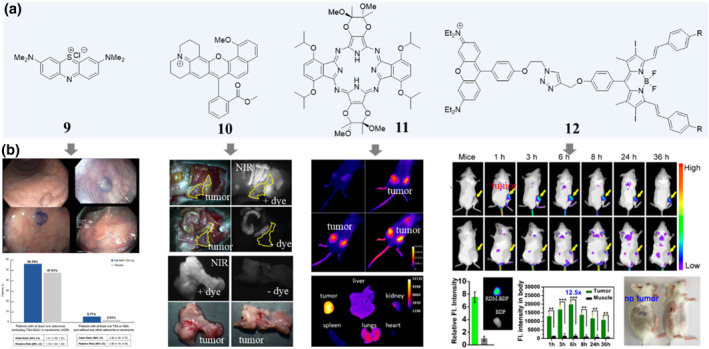
Chemical structures (a) of some selected tumor‐seeking dyes and their corresponding in vivo performances (b). Methylene blue (compound 9) was used for adenoma detection in patients undergoing colorectal cancer screening, adapted from, ref^[23]^ Copyright © 2019, by the AGA Institute. In vivo and ex vivo fluorescence imaging shows that compound 10 selectively accumulates in PDAC tumor tissue, adapted from, ref^[29]^ Copyright © 2019 The authors. Accumulation of compound 11 in MDA‐231 tumors over time and its biodistribution, adapted from, ref^[25]^ Copyright © 2009 National Academy of Sciences. In vivo and ex vivo fluorescence imaging with compound 12 in 4T1 tumor tissue and its treatment effect, adapted from, ref^[27]^ Copyright © 2018 American Chemical Society.

Since the discovery and later successful commercial usage of mauveine, many dyes have been constructed. Among these dyes, azo dyes play an important role and have been widely used in many fields. Evans blue (EB) dye is one of the most outstanding azo dyes. EB has a high affinity for serum albumin and has been an important tool in many physiologic and clinical investigations. For example, EB can be used for estimation of blood volume, determination of cardiac output, assessment of the myocardial area at risk and assessment of infarcted myocardium, assessment of vascular permeability, and detection of lymph nodes.^[20]^ Dyes binding to serum albumin have enhanced blood half‐life and thus have more opportunities to accumulate in tumor tissues. Therefore, EB is also an tumor‐seeking dyes and widely used for the diagnosis and identification of tumors.^[21]^ Because the structure of EB has many modification sites and some modifications still maintain tumor‐seeking property, EB can be conveniently tailored into tumor targeting groups used for targeting imaging. For example, connecting a PET isotope to the tailored EB results in a tumor targeted PET tracer.^[22]^ Similarly, methylene blue (**9**, Figure 3), a historical organic dye that is FDA‐approved for methemoglobinemia and an antidote to cyanide poisoning previously, has been explored and clinically approved as an agent for use in colonoscopy procedures due to its ability to selectively stain and visualize certain structures within the colon.^[23]^ It has been used to help delineate the margins of colorectal tumor lesions. Selective staining of the lesion can provide a clear visual contrast, assisting the endoscopist in determining the extent of the abnormality and guiding appropriate treatment or resection.^[24]^

As optical imaging agents as well as photosensitizers for photodynamic therapy (PDT), porphyrins, phthalocyanines, and related tetrapyrroles have attracted extensive attention.^[25]^ Porphyrazines are a class of macrocycles that combine some of the features of porphyrins and phthalocyanines while exhibiting distinctive properties of their own. For example, taking advantage of the lipophilic of low‐density lipoprotein and receptor‐mediated endocytosis (compound **11**, Figure 3), a chiral porphyrazine with strong NIR fluorescence emission shows preferential accumulation in the tumor tissue.^[25]^ Further enhanced phototoxicity for PDT may be achieved by simply inserting metal in the core of compound **6** or its derivatives. Taken together, this porphyrazine may provide an interesting platform for the development of optical contrast and even therapeutic agents due to its tumor‐seeking ability and ideal optical properties.

Compared with normal tissues, tumors have a lot of abnormities, such as different cell types, different microenvironments, etc. For example, researches show that cancer cells have higher magnitude of membrane potential than normal cells, thus compounds possess permanently delocalized positive charge such as rhodamine derivatives may preferentially accumulate in tumors.^[26]^ Recently, a classical rhodamine structure was connected with a long‐wavelength diiododistyrylbodypy and the result compound **12** (Figure 3) has some interesting properties such as tumor targeting potency, increased light‐harvesting ability and enhanced ^1^O_2_ generation.^[27]^ This is a novel strategy to develop structure inherent targeting therapeutic agents and maybe adopted to improve the performance of other structures such as phthalocyanines, porphyrins or porphyrazines. For pancreatic ductal adenocarcinoma (PDAC), the hypovascular and fibrotic tumor microenvironments are the two main features.^[28]^ Therefore, for the development of PADC seeking dyes, the molecule size, lipophilicity, solubility, and ionization state should be carefully considered. After systematical modifying a class of red and near‐infrared fluorescent dyes that exhibit a field‐effect‐derived redshift and careful screening, one nice seminaphthofluorophore (compound **10**, Figure 3) was found to accumulate selectively in the PDAC tumor. With the maximum emission wavelength about 622 nm, compound **10** enables visualization of four levels of the structure, including the subcellular organelles, individual cells, resected tissue and whole organ.^[29]^

Just like a drug molecule, the structure of dye molecules plays a critical role in their ability to selectively target and bind to specific biological structures. The presence of particular functional groups, aromatic rings, and charged moieties can confer inherent targeting capabilities to dyes, allowing them to preferentially accumulate in tumor tissues. Understanding these structural features and how they influence the targeting properties of dyes is essential for designing effective structure inherent tumor‐seeking dyes used for biosensing and diagnostics. As the field of dye chemistry continues to advance, further innovations in molecular engineering will undoubtedly lead to even more sophisticated and selective tumor‐targeting dyes with enhanced capabilities.

### 2.2 Ligand mediated tumor‐seeking dyes

Selective targeting of dyes to specific tumor tissue is a crucial aspect of their utility in tumor diagnostics and therapy as well as imaging guided surgery. While some dyes can exhibit inherent targeting capabilities based on their molecular structure, as discussed above, another important approach is the conjugation of dyes to targeting ligands. These ligands are molecules that can bind with high affinity and selectivity to receptors, proteins, or other biomolecular targets of interest. By covalently attaching a dye to such a targeting ligand, the resulting dye‐ligand conjugate can be directed to accumulate at the desired site of interest, allowing for enhanced specificity and contrast in biological imaging and analysis. The choice of both the dye and the targeting ligand is critical in optimizing the performance of these ligand‐mediated targeting systems. In the following sections, we will explore the design considerations and various examples of ligand‐targeted dyes and their applications.

#### 2.2.1 Small molecule mediated tumor‐seeking dyes

**FIGURE 4:**
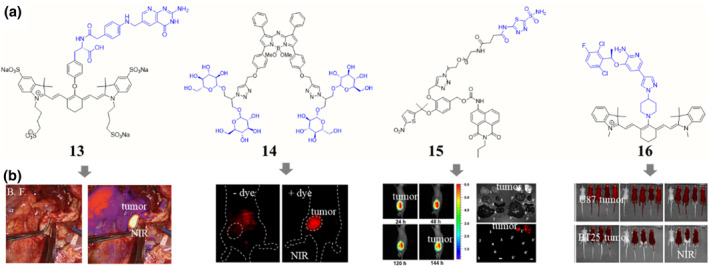
Chemical structures (a) of some selected small molecule mediated tumor‐seeking dyes and their corresponding in vivo performances (b). Visualization of ovarian cancer lesions in the right paracolic gutter using normal white light and NIR fluorescence imaging following compound 13 injection, adapted from, ref^[35c]^ Copyright © 2022 by American Society of Clinical Oncology. NIR fluorescent image of mouse bearing 4T1 orthotopic breast cancer prior to injection and following injection of compound 14, adapted from, ref^[38b]^ Copyright © 2023 American Chemical Society. In vivo and ex vivo fluorescence imaging xenograft tumor after intravenous injection of compound 15, adapted from, ref^[41]^ Copyright © 2021 American Chemical Society. In vivo fluorescence imaging of dye signal localization in intravenously injected tumor‐bearing mice with compound 16, adapted from, ref^[42]^ Copyright © 2023 The Authors. Blue, targeting moieties.

Incorporating both a fluorescent dye and a small molecular targeting moiety within a single molecule can result in a tumor‐seeking dye.^[30]^ Common targeting ligands include metabolic substrates, receptor‐binding pharmacophores, or other small molecules that exhibit high‐affinity interactions with the tumor tissue.^[31]^ Folate receptor *α* (FRα) has a high affinity for folates and is overexpressed in several solid tumors, such as ovarian, triple‐negative breast and lung cancers, making it a promising target for tumor‐seeking dyes development.^[32]^ Fluorescent folate conjugates which have tumor‐seeking ability can improve the surgical resection of FRα‐expressing cancers when used intraoperatively.^[33]^ The fluorophore scaffolds used for conjugation can be varied, and the in vivo performances of these conjugates are different.^[34]^ Encouragingly, one of the fluorophore‐folate conjugates pafolacianine (**13**, Figure 4), which shows high tumor‐seeking ability has been approved by the FDA for clinical application.^[35]^ Similarly, glucose is a common metabolic substrate for living organisms, and due to the fact of abnormal metabolism for tumor tissues, the demand for glucose is more vigorous for tumor tissues.^[36]^ Just like fluorine‐modified glucose fluorodeoxyglucose [^18^F]FDG retains tumor targeting thus is widely used clinically for tumor imaging,^[37]^ connecting one or several glucose structures to a fluorophore may endow its tumor‐seeking ability. For example, one increasingly common application is the use of fluorescent glucose analogs, such as 2‐NBDG and Azo‐BODIPY‐glucose conjugate (**14**, Figure 4), to image tumor metabolism in vivo.^[38]^ These small molecule dyes take advantage of the elevated glucose uptake and glycolytic activity of many cancer cells, allowing visualization of primary tumors as well as metastatic lesions.^[39]^

Beside the metabolic substrates such as folate and glucose, some clinical drugs and preclinical small molecules can also be used for tumor‐seeking dyes development.^[40]^ For example, the combination of a small‐molecule compound such as pharmacophore acetazolamide (**15**, Figure 4),^[41]^ crizotinib (**16**, Figure 4),^[42]^ indomethacin,^[43]^ histone deacetylase 6 inhibitor^[44]^ and a fluorophore maintains the tumor‐seeking ability. These conjugates can be used for in vivo tumor imaging with low background signal or high selective tumor therapy. Meanwhile, careful selection of the dye and targeting components, as well as optimizing physicochemical properties like charge and lipophilicity, are critical for developing small molecule‐based targeting dyes with the desired specificity, brightness, and photostability.

#### 2.2.2 Peptide mediated tumor‐seeking dyes

**FIGURE 5:**
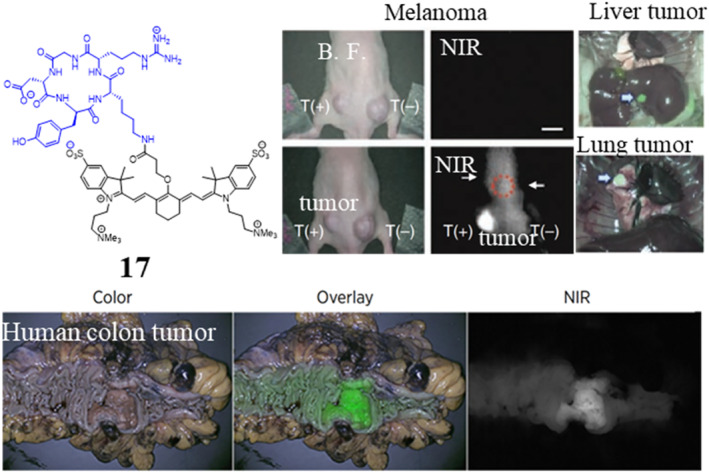
Chemical structures of the tumor‐seeking dye compound 17 and its corresponding in vivo performances. Intraoperative melanoma, liver tumor, and lung tumor detection using compound 17 (upper), adapted from, ref^[48a]^ Copyright © 2013, Springer Nature America, Inc. Gross macroscopy of a colon tumor in a patient treated with compound 17 (below), adapted from, ref^[49]^ Copyright © 2020 AACR. Blue, targeting peptide.

Peptides offer several advantages over small molecules, including their ability to target specific cell surface receptors with high affinity and selectivity or customizable sequences. By conjugating peptide ligands to fluorescent dyes, tumor‐seeking dyes can be productively created.^[45]^ One prominent example is the RGD (arginine‐glycine‐aspartic acid) peptide, which targets the αvβ3 integrin that is upregulated on proliferating endothelial cells during tumor angiogenesis and cancer cells of a lot of cancer cell lines.^[46]^ Therefore, targeting tumor vasculature or tumor cells by RGD‐based strategies has become a promising approach for delivering anticancer drugs or contrast agents for tumor therapy and diagnosis. As a result, diverse RGD peptide ligands have been developed. Among them, a class of cyclic RGD penta‐peptide analogs including c(RGDfV) and c(RGDfK) is preeminent for its high receptor binding affinity and specificity.^[47]^ Many RGD peptide‐fluorophore conjugates (e.g., cRDG‐ZW800‐1, **17**, Figure 5) retain the tumor‐seeking ability and have been used to visualize tumor vasculature and monitor anti‐angiogenic therapies in xenograft and genetic mouse models of cancer.^[48]^ Some of these RGD‐dye conjugates have been tested in human.^[49]^ Similarly, cell‐penetrating peptides,^[50]^ such as the p28 peptide derived from azurin,^[51]^ have also been used to deliver fluorescent dyes into the tumor tissue. These peptide‐dye conjugates can not only light up the tumor but also intraoperatively identify tumor cells in the margin to increase the possibility of complete resection.^[52]^ Besides, some peptides discovered from the phage display technology, such as the esophageal neoplasia targeted ASYNYDA peptide EGFR targeted QRHKPRE peptide and ErbB2 targeted KSPNPRF peptide, have been successfully used for labeling fluorophores. These peptide‐fluorophore conjugates show high tumor‐seeking ability and low toxicity.^[53]^ Clinical results in humans show that they are useful for guiding tissue biopsy and for improving visualization of flat and subtle pre‐malignant lesions.^[54]^ Targeting peptides that bind to receptors overexpressed on tumor cells, such as the somatostatin receptor, have also been explored. Conjugating these peptides to near‐infrared fluorescent dyes has enabled in vivo imaging of primary tumors and metastatic lesions in preclinical cancer models.^[55]^ Of course, many other receptor targeted peptides are also excellent candidates for constructing tumor‐seeking peptide‐fluorophore conjugates.^[56]^ For example, BLZ‐100, composed of a modified chlorotoxin peptide and a covalently conjugated ICG moiety, shows high selectivity accumulation in tumor and is well tolerated at all dose levels tested in human study.^[57]^ OTL78, composed of a high‐affinity PSMA‐targeting peptide DUPA and a covalently conjugated hydrophilic Cy7 moiety, shows high selectivity accumulation in prostate cancer both in preclinical and in clinical patient study.^[58]^

Beyond receptor mediated peptide targeting mentioned above, some tumor microenvironment mediated peptides, such as pH (low) insertion peptide (pHLIP), also been widely used to conjugate with fluorophore to fabricate tumor‐seeking dyes. Low pH is a hallmark of solid tumor at both very early and advanced stages of tumor development.^[36]^ Therefore, light up an acidic environment might reveal possible sites of tumor and targeted delivery to these tissues might improve the performance of tumor treatment. pHLIP is soluble in water and bound to the surface of a membrane and inserted across the membrane as an *α*‐helix at acidic pH, thus accumulated in the tumor tissue.^[59]^ Various tumor‐seeking dyes have been constructed by conjugating pHLIPs to fluorophores and used for different in vivo applications.^[60]^

As the peptide engineering toolkit continues to expand, the ability to rationally design peptide‐dye conjugates with optimal targeting, pharmacokinetics, and signal‐to‐noise properties will enable increasingly sophisticated applications of peptide‐mediated targeting for in vivo diagnostics and molecular imaging.

#### 2.2.3 Protein or nucleic acid mediated tumor‐seeking dyes

In addition to small molecules and peptides, both protein‐ and nucleic acid‐based targeting moieties have demonstrated utility for delivering fluorescent dyes to tumor tissues. Antibodies and antibody fragments, with their exquisite targeting specificity, have been extensively explored as carriers for optical imaging dyes. For example, endothelial growth factor receptor (EGFR) is overexpressed in many types of tumors and has been frequently used as a target in tumor imaging; monoclonal antibodies targeting EGFR such as cetuximab, bevacizumab, and panitumumab have been approved for clinical applications. Meanwhile, the cyanine dye IRDye800CW was used to conjugate with these antibodies. The resulting antibody‐fluorophore conjugates, cetuximab‐800CW,^[61]^ bevacizumab‐800CW,^[62]^ panitumumab‐800CW,^[63]^ have demonstrated excellent tumor‐seeking ability and safety in clinical trials. Besides, tumor biomarker carcinoembryonic antigen (CEA) which overexpresses in more than 95% of digestive tumors is also a valuable target for antibody‐fluorophore development. SGM‐101 is an anti‐CEA receptor‐targeted antibody coupled with a fluorophore Cy5 with an emission wavelength of 700 nm. SGM‐101 has also been demonstrated outstanding tumor‐seeking ability and safety in pancreatic cancer and colorectal lung metastases clinical trials.^[64]^ Beside these traditional dyes with emission within 800 nm, fluorophore with emission beyond 1000 nm can also be conjugated with antibody and the resulting in vivo tumor images have better resolution and deeper tissue penetration depth.^[65]^ At the same time, we should keep in mind that attaching a small molecule such as a fluorophore to an antibody often significantly impacts the properties of the resulting conjugate.^[66]^ Therefore, more balanced fluorophores with impressive optical performance and little influence on the antibody are urgently needed. In addition to the antibodies mentioned above, some protein, such as transferrin, can also be used as tumor targeting protein for tumor‐seeking protein‐fluorophore development.^[67]^

Similarly, aptamers—short, synthetic nucleic acid ligands—have emerged as an alternative tumor targeting scaffold. Aptamer‐dye conjugates have been developed to image a range of tumor cellular receptors and molecular signatures.^[68]^ Compared to small peptides, larger protein and nucleic acid probes can offer enhanced binding affinities and prolonged circulation times in vivo. However, their increased size and complexity can also present challenges, such as suboptimal tissue penetration, potential immunogenicity, and more complex manufacturing requirements. Strategies to address these limitations, such as engineering smaller antibody fragments or stabilizing aptamer structures, will help to expand the utility of protein‐ and nucleic acid‐based targeting dyes.

## 3 NERVE‐SEEKING DYES

Nerve‐seeking dyes are a class of fluorescent molecules that possess a high affinity for nerve tissues, enabling their visualization and study in living organisms. These dyes have become indispensable tools in the field of neuroscience, allowing researchers to track neuronal pathways and investigate the structure and function of the nervous system in vivo. Roughly, nerve‐seeking dyes can be divided into two kinds, namely structure inherent nerve‐seeking dyes and ligands mediated nerve‐seeking dyes. The unique ability of nerve‐seeking dyes to selectively label nerve tissues stems from their strong interaction with lipid‐rich cellular membranes, particularly those found in myelin sheaths and axonal projections. Besides, endoneurium is also a targeting site for nerve‐seeking dyes.^[69]^

### 3.1 Structure inherent nerve‐seeking dyes

**FIGURE 6:**
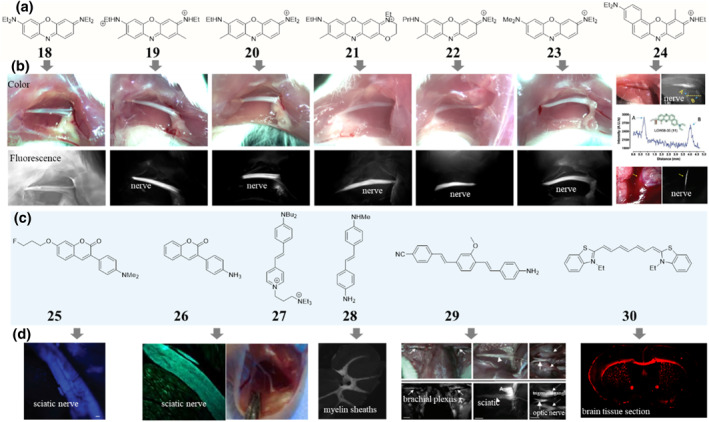
Chemical structures (a), (c) of some selected nerve‐seeking dyes and their corresponding in vivo performances (b), (d). Photographs and fluorescence images of NIR oxazine derivativesafter systemic administration, adapted from, ref^[70b]^ Copyright © 2020 The Authors, some rights reserved; exclusive licensee American Association for the Advancement of Science. compound 24 fluorescently highlighted brachial plexus nerves and buried iliac nerve visualization through pooled blood, adapted from, ref^[70e]^ Copyright © 2023 The Authors. Image of sciatic nerves following injection of compound 25, adapted from, ref^[71c]^ Copyright © 2011 American Chemical Society. compound 26 staining of tissue sections containing sciatic nerve, adapted from, ref^[71a]^ Copyright © 2010 Wang et al. In situ staining of myelin sheaths in the cerebellum of mouse brain with compound 8, adapted from, ref^[73]^ Copyright © 2008 American Chemical Society. Image‐guided surgery using compound 29 in rats, adapted from, ref^[74a]^ Copyright © 2011 Decker Publishing. In vitro staining of free‐floating brain tissue sections of wild‐type mice with compound 30, adapted from, ref^[75a]^ Copyright © 2011 The authors.

Staining the nerve selectively is a long‐standing pursuit of many researchers. Inspired by the hematoxylin and eosin (H&E) stain for tissue and luxol fast blue (a copper phthalocyanine) stain for myelin. Many small molecular structure inherent nerve‐seeking dyes have been discovered, including oxazine,^[70]^ coumarin,^[71]^ styryl pyridinium,^[72]^ stilbene,[^3^, ^73^] distyrylbenzene^[74]^ and cyanine^[75]^ fluorophores (Figure 6). Oxazine dyes are a class of compact and rigid fluorophores that contain an oxazine structure with moderate NIR fluorescence emission. Classical oxazine dyes, such as Nile red and Nile blue, have been widely used for biological stains. The first nerve‐seeking oxazine structure (**19**, Figure 6a) was discovered from judiciously ex vivo and in vivo screening a dye library containing four oxazine dyes and a rhodamine fluorophore.^[70a]^ Presumably, compound **19** likely targets myelin or a closely associated molecule. After intravenous injection, compound **19** shows remarkable nerve signals against both muscle and adipose tissues. Meanwhile, despite the very similar structures, compound **18** (Figure 6a) exhibited a drastically different biodistribution pattern resulting in minimum nerve‐specific contrast. The long wavelength and distinct biodistribution of these two oxazines have provoked the curiosity of researchers. Subsequently, the oxazine fluorophore was been systemically modified and a library of 64 oxazine structures derived from compound **18** and compound **19** were constructed. Through a comprehensive screening process involving topical and systemic administration in rodent models, four innovative near‐infrared (NIR) fluorophores (**20**, **21**, **22**, and **23**, Figure 6a) that selectively bind to nerve tissues were identified (Figure 6b). In a series of fluorescence‐guided surgical procedures in swine models, these nerve‐seeking dyes demonstrated the ability to clearly delineate and differentiate buried lumbar and iliac nerves, which are challenging to visualize under conventional white light. Notably, the use of these nerve‐specific fluorescent dyes allowed the surgical team to easily identify and avoid a small nerve branch that would have been at high risk of accidental transection using standard white light visualization alone.^[70b]^ On the basis of above works, incorporating a benzene into the oxazine structure resulting in a library of 12 benzo[c]phenoxazine fluorophores with emission beyond 750 nm that are fully compatible with the 800 nm imaging channel already present in the vast majority of clinical FGS systems. After in vivo nerve‐specificity screenings, 7 out of 12 exhibited positive nerve contrast. Importantly, among the 7 nerve‐seeking dyes, compound **24** (Figure 6a) demonstrated the remarkable ability to accurately delineate small nerve branches with an average diameter of approximately 200 microns (Figure 6b). This size range is noteworthy, as it is equivalent to the lower limit of human prostatic nerve diameter, which is generally 200 microns or greater.^[70e]^

Beside oxazine dyes, coumarin (**25**, **26**) and stilbene (**28**) are two interesting nerve‐seeking dyes. Though the wavelength of coumarin and stilbene are both generally short thus is not ideal for in vivo optical based applications, radioisotope ^18^F can be conveniently introduced to the 7‐site of coumarin and the stilbene core structure, resulting in nerve‐seeking dyes as well as a PET tracers.[^3^, ^71^] Since PET imaging has become a routine clinical diagnostic strategy without penetration depth limitation, these nerve‐seeking dyes may be used for diagnosing myelin‐related diseases and determining the efficacy of therapies. For distyrylbenzenes (**29**), although more than 200 analogs have been synthesized and structure‐activity relationship (SAR) verified the overall configuration of the pharmacophore was the dominant feature that influenced nerve‐specificity, none of which emits beyond the visible region. Therefore, none of the current distyrylbenzenes are ideal for nerve‐specific image‐guided surgery or other in vivo based application.^[74c]^ From the point of view of wavelength, among these structure inherent nerve‐seeking dyes, the 3,3‐diethylthiatricarbocyanine iodide (DBT, **30**, Figure 6c) maybe has the longest emission wavelength. DBT easily enters the brain and selectively binds to myelin sheaths in animal models.^[75a]^ However, for nerve‐seeking cyanine dyes, up to now, there is still a lack of structure diversity and more convincing data about toxicology, pharmacokinetics (PK), and pharmacodynamics (PD) for driving further research.

From the view of in vivo optical‐based applications, among the fluorophores reported for nerve‐seeking, including oxazine, coumarin, styryl pyridinium (**27**), stilbene, distyrylbenzene and cyanine fluorophores, oxazine fluorophores may possess better potential for translation due to their NIR emission and variable structures. Of course, more solid data about toxicology, PK, and PD for any potential compounds are welcomed.^[70c,70g]^

### 3.2 Ligand mediated nerve‐seeking dyes

Compared with tumor, the tight and conserved nervous tissue is more difficult to target. Up to now, only a small number of peptides and proteins have been reported to have nerve‐seeking potential. Utilizing a phage display and in vitro selection approach against excised mouse peripheral nerve tissue, a nerve‐specific peptide NP41 was discovered. This peptide was then conjugated to fluorescent dyes, creating two nerve‐seeking dyes, FAM‐NP41 and Cy5‐NP41. Both dyes demonstrated specificity for various peripheral nerves, including the brachial plexus, sciatic, phrenic, and dorsal cutaneous nerves, when validated in mouse models.^[76]^ Microscopy revealed that Cy5‐NP41 primarily localized to the epineurium rather than myelin or axonal membranes. Building on this success, a next‐generation nerve‐targeting peptide, HNP401, which showed similar nerve‐specific contrast but improved signal intensities in rodents compared to FAM‐NP41 was further developed.^[77]^ This HNP401 probe is currently undergoing clinical trials for intraoperative use during head and neck surgery, highlighting the potential of these nerve‐specific peptides for clinical translation.^[78]^ In addition to the epineurium, the axon is another promising target within peripheral nerve bundles. The Hsp1a peptide, derived from the venom of the Peruvian tarantula, is a potent inhibitor of the voltage‐gated sodium channel Na_v_1.7, which is expressed in both human and mouse tissues. The Hsp1a peptide was conjugated to the fluorophore BODIPY, creating the fluorescently labeled nerve‐seeking dye Hsp1a‐FL. Following systemic administration, Hsp1a‐FL demonstrated nerve‐specificity in mouse sciatic nerves. Further histological and microscopy analysis confirmed the Na_v_1.7‐specific binding of this probe to the axons.^[79]^ To enable near‐infrared nerve visualization, the team subsequently switched the labeling fluorophore from BODIPY to Cy7.5 and bacteriochlorin.^[80]^ The utility of this nerve‐targeting peptide has also been expanded to encompass other imaging modalities beyond fluorescence, broadening its potential clinical applications.^[81]^ Separately, using truncated homotypic myelin protein zero (P0) peptide sequences as targeting peptides for the peripheral nervous system has also been explored. Through screening fluorescently labeled P0 peptide candidates, Cy5‐P0_101‐125_ as a potent nerve‐specific agent for myelin under various in vitro, ex vivo, and in vivo conditions was identified. Like the other peptide‐based probes, Cy5‐P0_101‐125_ can also be systemically administered to produce nerve‐specific contrast within hours.^[82]^

While antibody‐fluorophore conjugates have been explored for nerve‐specific imaging, their utility is limited in vivo due to the tight blood‐nerve barrier (BNB) junction, which generally prevents the crossing of macromolecules. As a result, there are relatively few successful preclinical examples of nerve imaging using protein‐based dyes. Gratifyingly, there are still some promising results with protein‐based nerve imaging agents. For example, the “Nervelight”, a recombinant human nerve growth factor (rhNGF) protein labeled with an 800 nm fluorophore, was able to show fluorescence in the rat sciatic, cavernous, facial, and axillary nerve tissues.^[83]^ The targeting mechanism was hypothesized to be the probe's affinity for the tropomyosin receptor kinase A (TrkA), which is an overexpressed, high‐affinity receptor for nerve growth factor (NGF) at the distal nerve endings. Similarly, an anti‐ganglioside monoclonal antibody labeled with Dylight® 550 dye also showed the ability to delineate intact motor, sensory, and autonomic nerve tissue fluorescence in mice 6 days after intraperitoneal injection.^[84]^

Nerve‐seeking fluorescent dyes have become valuable tools for in vivo nerve visualization, enabling enhanced surgical precision and reduced iatrogenic nerve injuries. The continued development and optimization of these dyes, leveraging ligand‐mediated targeting strategies and state‐of‐the‐art fluorophore design, promise to further improve patient outcomes across a wide range of clinical applications, from neurosurgery and urology to pain management and beyond. As these nerve‐specific fluorophores become more sophisticated and clinically translatable, they are poised to revolutionize the way surgeons approach delicate nerve‐sparing procedures, ultimately enhancing the quality of life for countless patients.^[85]^

## 4 BONE‐SEEKING DYES

**FIGURE 7:**
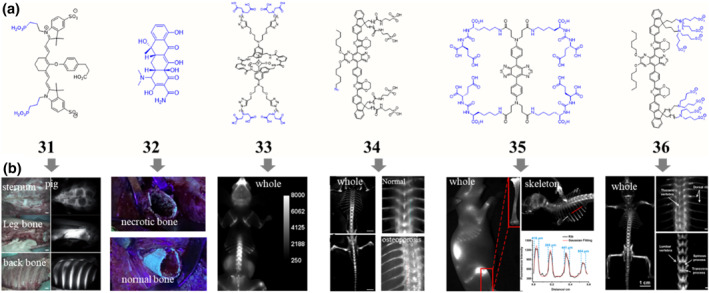
Chemical structures (a) of some selected bone‐seeking dyes and their corresponding in vivo performances (b). Bone tissue imaging using compound 31, adapted from, ref^[87]^ Copyright © 2014, Wiley‐VCH Verlag GmbH and Co. Intraoperative photographs of the surgical field using an ultraviolet lamp of a patient that had been receiving minocycline, adapted from, ref^[93]^ Copyright © 2021 The authors. NIR fluorescence imaging of skinless mice treated with compound 33, adapted from, ref^[95a]^ Copyright © 2013 American Chemical Society. Fluorescence imaging of the whole skeletal system and osteoporotic mice using 34, adapted from, ref^[96]^ Copyright © 2021 American Chemical Society. In vivo and ex vivo fluorescence imaging mice with the administration of compound 35, adapted from, ref^[97c]^ Copyright © 2023 American Chemical Society. Fluorescence imaging of the whole skeletal system and some pivotal skeletal system using compound 36, adapted from, ref^[98]^ Copyright © 2023 The author. Blue, targeting moieties.

Bone‐targeting dyes are a class of fluorescent compounds that have a strong affinity for hydroxyapatite, the primary mineral component of bone. These dyes are widely used in various in vivo applications, including bone imaging, fracture detection, and the evaluation of bone remodeling processes.^[86]^ One class of the most commonly used bone‐targeting dyes are the phosphonate‐based fluorophores. Phosphonates, such as pamidronate and alendronate, have high affinity for hydroxyapatite, allowing them to be used for the targeted delivery of imaging agents or therapeutic payloads to bone tissue. Various NIR fluorophores have been modified with phosphonate to get the bone‐seeking dyes. For example, Cy5‐phosphonate,^[87]^ Cy7‐phosphonate (**31**, Figure 7),[^87^, ^88^] Cy7.5‐phosphonate^[89]^ and xanthene‐phosphonate^[90]^ all show bone‐seeking capability. They can bind to growing regions of the bone or accumulate in the osteolytic lesion, allowing surgeons to perform the surgery with the guidance of NIR fluorescence. Meanwhile, data show that the overall molecular net charge and hydrophobicity of targeted fluorophores play a compelling role in the biodistribution, blood half‐life, organ accumulation and metabolism route of intravenously administered contrast agents.^[88b,88c]^ Another interesting strategy is biorthogonal pretargeting. The *trans*‐cyclooctene‐modified bisphosphonate was administered previously, and then tetrazine‐derived cyanine dye was injected. The click chemistry between tetrazine and *trans*‐cyclooctene make the cyanine fluorophore become a bone‐seeking dye.^[91]^ Meanwhile, recent studies have indicated that the use of bisphosphonate‐based fluorescent probes can inadvertently induce apoptosis in osteoclasts, which may potentially interfere with the normal physiological processes that the imaging technique aims to capture and monitor.

Another prevalent bone‐seeking dye is tetracycline (**32**, Figure 7), a clinically used broad‐spectrum antibiotic that also has a high affinity for calcium‐rich structures, such as mineralized bone. Tetracycline and its derivatives, including doxycycline and minocycline, have been extensively studied for their ability to label bone surfaces and enable the visualization of bone formation, resorption, and mineralization dynamics.^[92]^ During septic hip revision surgery, tetracycline can be used as an intraoperative tool to debride necrotic bone.^[93]^ Nevertheless, the maximum emission wavelength of tetracycline and its derivatives is about 530 nm, which is not ideal for fluorescence guided surgery due to the strong tissue autofluorescence in the visible region. Therefore, tetracycline‐NIR fluorophore conjugate is another choice. For example, the IRDye 800CW was conjugated with tetracycline and the resulting conjugate can be used as a general marker of skeletal features as well as an indicator of the bone mineralization and remodeling processes.^[94]^

In addition to phosphonates and tetracyclines, iminodiacetate,^[95]^ azide,^[96]^ peptide (**35**),^[97]^ sulfonate,^[98]^ and other moieties^[99]^ have been successfully demonstrated to have bone‐seeking properties (Figure 7). Although iminodiacetate and sulfonate have an inherently lower bone affinity than bisphosphonates, fluorophores modified with a dendritic molecular structure with four iminodiacetate groups (**33**) or eight sulfonate groups (**36**) show good bone‐seeking capability.[^95^, ^98^] Interestingly, the dendritic sulfonate‐based fluorophore exhibits significant differences between osteoporosis and normal bone with a higher accumulation in osteoporosis bone, which may be attributed to osteoclast activity. Encouraged by 3′‐azido‐3′‐deoxythymidine accumulates in the bone,^[100]^ azide group modified NIR‐II dye (**34**, Figure 7) has been verified with bone‐seeking capacity.^[96]^ The azide group equips compounds bone‐seeking capacity opening a new window for the biological application of the azide group, and providing another attractive design strategy for achieving in vivo bone targeting.

Collectively, bone‐targeting dyes have become an indispensable tool for in vivo bone research and clinical diagnostics, enabling the real‐time visualization and quantification of various bone‐related processes and pathologies. Ongoing research is focused on developing new and improved compounds with enhanced specificity, sensitivity, and biocompatibility. As the field continues to evolve, the development of novel bone‐seeking dyes and their integration with advanced imaging technologies promise to yield new insights and improved clinical outcomes in the management of bone‐related disorders.

## 5 OTHER TISSUE‐SEEKING DYES

**FIGURE 8:**
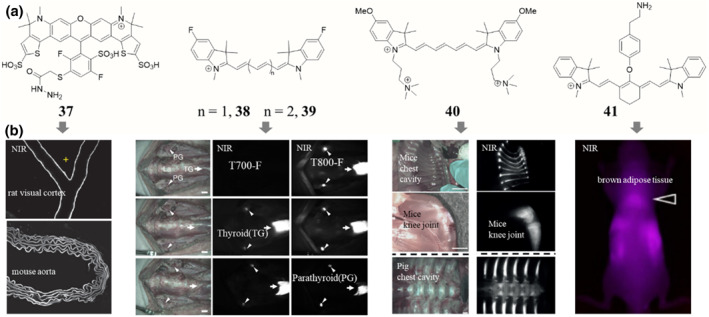
Chemical structures (a) of some selected tissue‐seeking dyes and their corresponding in vivo performances (b). NIR fluorescence images from the rat visual cortex and a mouse aorta after labeling with Alexa Fluor 633 (compound 37) in vivo, adapted from, ref^[101]^ Copyright © 2012 Springer Nature America, Inc. In vivo NIR imaging of parathyroid and thyroid glands in pigs with compounds 38 and 39, adapted from, ref^[104]^ Copyright © 2015 Springer Nature America, Inc. Fluorescence imaging of cartilage with compound 40, adapted from, ref^[105]^ Copyright © 2015 Wiley‐VCH Verlag GmbH and Co. Fluorescence imaging shows that compound 41 selectively accumulates in brown adipose tissue, adapted from, ref^[106]^ Copyright © 2022 The authors.

In addition to the well‐established applications of tissue‐specific dyes for targeting tumors, nerves, and bone, the field of in vivo optical imaging has expanded to encompass a diverse range of tissue‐seeking fluorescent probes. These innovative dyes enable the non‐invasive visualization and tracking of an increasingly broad spectrum of biological structures and processes within living organisms. One particularly exciting area is the development of dyes that selectively label the vasculature. By rendering the intricate network of blood vessels fluorescent, these dyes allow for the real‐time monitoring of angiogenesis, vascular permeability, and blood flow dynamics—all of which are critical for understanding both normal physiological and pathological conditions. For instance, Alexa Fluor 633 (**37**, Figure 8) has been successfully used to monitor changes in arterial dynamics in response to sensory stimulation for the reason that it electively bound elastin fibers in arterial walls in mouse, rat, cat and macaque monkey brains but did not label veins, capillaries, neurons, astrocytes or pericytes.^[101]^ Meanwhile, continuously real‐time monitoring of dynamic vascular processes, such as ischemic reperfusion, thrombolysis, opening and recovery of the blood brain barrier (BBB) can be conveniently performed with a NIR fluorophore which has a long blood half‐life.^[102]^

Besides, the endocrine glands, such as thyroid and parathyroid glands, which secrete hormones that regulate blood calcium levels, are tissues of substantial clinical importance. Interestingly, through carefully modulating the lipophilicity, electronic contributions, and charge characteristics of near‐infrared cyanine dyes, selective and enhanced uptake within the endocrine system can be achieved, thus enabling the selective labeling and visualization of these biologically important structures.^[103]^ For example, pentamethine and heptamethine cyanine moieties (**38**, **39**, Figure 8), have enabled the selective localization of near‐infrared fluorescent dyes in the thyroid and parathyroid glands, respectively.^[104]^ These structure‐inherent tissue‐seeking dyes provide surgeons with explicit image guidance during head and neck surgery. Similarly, the use of positively charged quaternary ammonium groups has facilitated the targeting of cartilage tissue with cyanine fluorophores (**40**, Figure 8) by taking the advantage of the negatively charged glycosaminoglycans present in cartilage.^[105]^

Brown adipose tissue (BAT) plays a crucial role in metabolism and thermoregulation and has been recognized as an attractive target for the treatment of conditions like obesity and diabetes. Researchers are exploring strategies to activate, increase, and “brown” white adipose tissue as potential therapeutic approaches. In this regard, noninvasive imaging of brown adipose tissue (BAT) using NIR fluorescence has emerged as a valuable tool in this field. Specifically, the NIR fluorescent dye IR‐786 and its derivatives (**41**, Figure 8) have demonstrated a remarkable ability to accumulate within BAT after intravenous administration. This allows for the accurate detection and quantification of BAT, irrespective of whether the subject is a tumor‐bearing or normal mouse model, which provides a powerful and minimally invasive approach to visualize and study the distribution and activity of BAT in various physiological and pathological conditions.^[106]^

Overall, tissue‐seeking fluorescent dyes have become invaluable tools for in vivo tissue visualization. The continued development of tissue‐seeking dyes, leveraging ingenious fluorophore design with inherent tissue‐seeking capability or ligand‐mediated targeting strategies, promises to advance a wide range of biological applications of NIR imaging in more tissues.^[107]^

## 6 CONCLUSION AND PERSPECTIVE

The strategic development of tissue‐specific contrast agents has emerged as a transformative approach in the field of in vivo imaging and theranostic applications. By harnessing the inherent chemical properties of the dye molecules or traditional ligand‐based targeting strategies, we can achieve highly selective labeling and visualization of a wide range of tissues, including tumors, nerves, bone, and beyond. The core principle underlying this success has been the rational tuning of physicochemical parameters, such as lipophilicity, charge, and structural motifs, to exploit the natural chemical recognition between the dye and the target tissue.^[108]^ Actually, there are some guidelines for the design of tissue‐seeking dyes. (1) Selectivity and affinity. The purposed dye should selectively bind to specific tissue types to minimize background interference. By optimizing chemical structures such as functional groups or linker moieties, the binding affinity can be enhanced. (2) Optical properties. Choosing dyes with excitation and emission wavelengths that minimize tissue autofluorescence and overlap with other signals. In other words, a longer wavelength may be better. Meanwhile, the dyes should have good brightness and stability. (3) Biocompatibility. The purposed dye should be safe enough for in vivo applications and better resistant to metabolic breakdown to prolong retention time in tissue. (4) Optimizing pharmacokinetics. The dye should have desirable pharmacokinetic profiles to ensure optimal tissue retention and clearance rates. (5) Validation and testing. A thorough testing benchmark against existing dyes should be conducted to identify unique advantages or improvements in relevant models to assess efficacy, safety, and real‐world applicability.

Looking to the future, the continued advancement of these tissue‐seeking dyes holds immense promise for revolutionizing in vivo diagnostics and monitoring. By providing highly specific and sensitive readouts of tissue function and pathology, these dyes can empower earlier disease detection, more accurate disease staging, and the real‐time evaluation of therapeutic interventions. In this regard, NIR dyes, especially NIR‐II dyes with high brightness and good tissue‐seeking ability have great potential since light penetrates tissues more easily in the region of longer wavelengths due to reduced scattering and absorption.^[109]^ Furthermore, the ability to multiplex several tissue‐specific dyes enables the simultaneous visualization of complex anatomical relationships, which could unlock unprecedented insights into integrative physiology and pathophysiology. Besides, the integration of tissue‐seeking dyes with complementary imaging modalities, such as MRI, CT, or ultrasound, could provide enhanced spatial resolution, anatomical context, and functional information, ultimately improving diagnostic capabilities.

As the field continues to evolve, researchers will undoubtedly uncover new design principles and chemical scaffolds to expand the repertoire of tissue‐specific contrast agents, addressing current limitations and unlocking new applications. The integration of advanced computational tools, such as artificial intelligence (AI) and machine learning, could revolutionize the design, screening, and analysis of tissue‐seeking dyes, accelerating the discovery and optimization of these imaging agents. Meanwhile, coupled with the rapid progress in imaging technologies, the future holds immense potential for these tissue‐seeking dyes to become indispensable tools for advancing biomedical research and transforming clinical practice.

While the rational design of tissue‐specific contrast agents has shown tremendous promise in preclinical studies, their successful translation to the clinic faces several key challenges that must be carefully addressed. One of the foremost concerns is ensuring the biocompatibility and safety of these dye molecules for human use. Rigorous toxicology assessments and pharmacokinetic evaluations will be essential to demonstrate the compounds' lack of systemic toxicity, immunogenicity, and off‐target effects. Particular attention must be paid to the clearance kinetics and tissue distribution to mitigate the risk of potential long‐term accumulation or undesirable biodistribution. Another critical hurdle is establishing the targeting specificity of these dyes with a high degree of confidence. While the preclinical data may show promising tissue selectivity, the complexity of human anatomy and physiology could result in unanticipated cross‐reactivity or off‐target binding. Robust validation studies, potentially including head‐to‐head comparisons with gold‐standard techniques, will be necessary to substantiate the dyes' ability to faithfully report on the target tissue of interest. The regulatory landscape for novel imaging agents also poses a significant challenge. Navigating the approval process with regulatory bodies, such as the FDA, EMA and NMPA, will require meticulous planning and extensive documentation to demonstrate the dyes' safety, efficacy, and compliance with stringent guidelines. This could involve conducting large‐scale clinical trials, gathering long‐term safety data, and fulfilling other regulatory requirements, all of which can be time‐consuming and resource‐intensive. Lastly, the successful translation of these tissue‐seeking dyes will depend on the ability to establish scalable and reproducible manufacturing processes. Ensuring consistent quality, purity, and batch‐to‐batch reproducibility will be crucial, as will the development of suitable formulations and delivery strategies that optimize the dyes' pharmacokinetic and targeting properties.

Overcoming these multifaceted challenges will require a concerted effort from interdisciplinary teams of chemists, biologists, clinicians, and regulatory experts. However, the potential rewards of successfully translating these innovative tissue‐specific contrast agents to the clinic are immense, offering the promise of transformative advancements in disease diagnosis, monitoring, and targeted treatment.

## CONFLICT OF INTEREST STATEMENT

The authors declare no conflicts of interest.

## Data Availability

Data openly available in a public repository.
